# Diagnostic Accuracy Comparison of Artificial Immune Algorithms for Primary Headaches

**DOI:** 10.1155/2015/465192

**Published:** 2015-05-04

**Authors:** Ufuk Çelik, Nilüfer Yurtay, Emine Rabia Koç, Nermin Tepe, Halil Güllüoğlu, Mustafa Ertaş

**Affiliations:** ^1^Department of Computer Engineering, Faculty of Computer and Information Science, Sakarya University, 54187 Sakarya, Turkey; ^2^Department of Neurology, Faculty of Medicine, Balikesir University, 10145 Balikesir, Turkey; ^3^Department of Neurology, Faculty of Medicine, Izmir University, 35530 Izmir, Turkey; ^4^Department of Neurology, Faculty of Medicine, Istanbul University, 34093 Istanbul, Turkey

## Abstract

The present study evaluated the diagnostic accuracy of immune system algorithms with the aim of classifying the primary types of headache that are not related to any organic etiology. They are divided into four types: migraine, tension, cluster, and other primary headaches. After we took this main objective into consideration, three different neurologists were required to fill in the medical records of 850 patients into our web-based expert system hosted on our project web site. In the evaluation process, Artificial Immune Systems (AIS) were used as the classification algorithms. The AIS are classification algorithms that are inspired by the biological immune system mechanism that involves significant and distinct capabilities. These algorithms simulate the specialties of the immune system such as discrimination, learning, and the memorizing process in order to be used for classification, optimization, or pattern recognition. According to the results, the accuracy level of the classifier used in this study reached a success continuum ranging from 95% to 99%, except for the inconvenient one that yielded 71% accuracy.

## 1. Introduction

Headache is a subjective topic concerning pain in the different parts of the head. The most commonly known version is migraine, and it is usually confused with other headaches. There are certain symptoms of headaches, and their diagnostic criteria are defined in* “The International Classification of Headache Disorders, Second Edition (ICHD-2)”* [1] by* “The International Headache Society (IHS)”* [2]. All of the criteria for headache diagnosis seem complicated because of the similarities between each diagnosis of headaches. Additionally, due to the lack of time spent on each patient at the hospitals, doctors may come up with a wrong diagnosis. The doctors (54% of the neurologists) in Turkey indicated that the error in the diagnosis of migraine is because of the density of patients in the hospitals [3]. So far, different algorithms such as decision tables [4] or various machine learning classifiers [5, 6] have been used by different researchers for headache diagnosis. What makes this study unique is the fact that it makes use of artificial immune system algorithms, which have recently been popular classifiers and these algorithms have not been used for the categorization of headache types.

**Figure 1:**
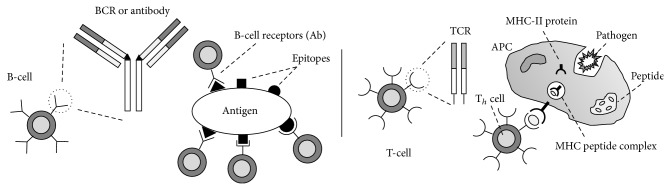
B-cell and T-cell pattern recognition of an antigen or pathogen.

Immune-inspired algorithms attracted the attention of computer scientists [7] because of their easy usage, flexibility, stable structure, and precise results. Basically, an immune system discriminates between the self (antibody) and nonself material (antigen, pathogen) in an organ. Mammals obtain immunity through a process of mutation, recognition, and proliferation and a process of memorizing when potentially harmful antigens stimulate the immune system by encountering T-cells or B-cells with an affinity calculation. T-cells and B-cells have receptors that bind to antigens from epitopes with their paratopes. The pattern recognition of T-cells and B-cells for an antigen or pathogen is illustrated in Figure 1.

## 2. Materials

We conducted this research on 850 patients with headache problems. Patients were from both sexes, were older than 15 years old, and lived in different cities from across Turkey.

For this study to work, patients needed to respond to the questions of doctors to keep medical records voluntarily. Doctors put this information into our web-based questionnaire hosted on http://www.migbase.com/ by using a tablet or computer. Therefore, informed consent was obtained from all patients, and the requirement for written, informed consent was waived by the investigational review board.

Doctors are supposed to enter their personal diagnosis into the system following the patient's completion of the questionnaire. The system does not allow doctors to see the diagnosis of the website before they enter their diagnosis. This system was set up by using the MySQL database and PHP language. Microsoft Excel was used to create the dataset from all of the records. We classified headaches by using WEKA Algorithm Implementation [8], which is a data-mining workbench software.

The questionnaire contains headache-related questions related to severity, localization, aggravation, characterization, nausea, photophobia, and aura symptoms as well as personal questions like gender, age, or smoking. The database has 40 attributes in total. The results of the software diagnosis and opinions of doctors can be accessed from the website [9].  Table 1 shows the neurologists' diagnoses.

According to the results, the migraine population is high, whereas the cluster type of headache is low, and there was only one patient who had no headaches. The algorithms implement the classification through a learning stage by using a training dataset that is derived from the research data. Thus, the variability of the training dataset is crucial for a successful classification.

## 3. Methods

### 3.1. Immunos Algorithms

**Figure 2:**
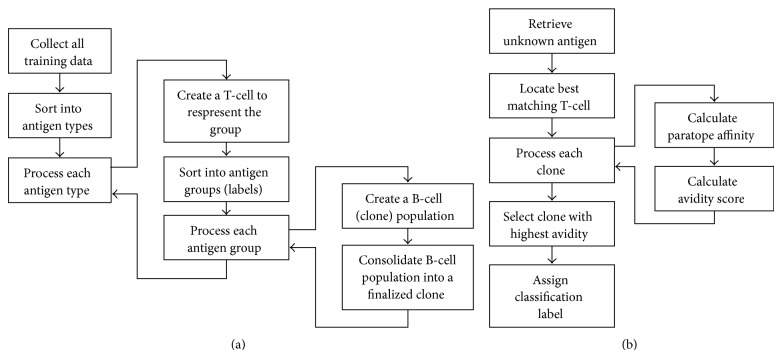
Immunos-81 algorithm. (a) General version of training. (b) Summary of the classification.

The first algorithm based on AIS, called “Immunos-81,” was developed by Carter [10] who is a medical doctor. He used T-cells to control the production of B-cells. All variables of the antigens transmitted to the system are stored in an amino acid library, and the T-cells use this library to recognize the new antigen. The T-cells create B-cells whose paratopes are matched to the epitopes of an antigen. Immunos-81 is an instance based classifier, and it has two implementations called Immunos-1 and Immunos-2. The algorithm training and classification process is shown in Figure 2.

**Figure 3:**
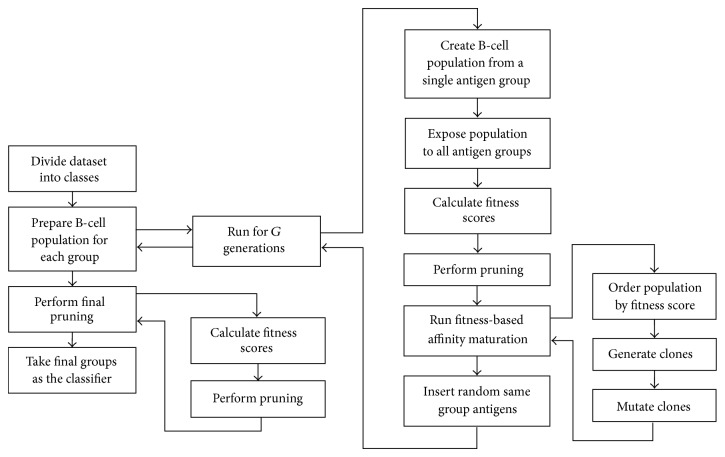
Immunos-99 algorithm. Classification and training process.

Brownlee [11] improved those classifiers by integrating cell proliferation and hypermutation techniques from other AIS-based algorithms and finally developed a new immunos algorithm called the Immunos-99 algorithm, which is shown in Figure 3.

### 3.2. Artificial Immune-Recognition System Algorithms

**Figure 4:**
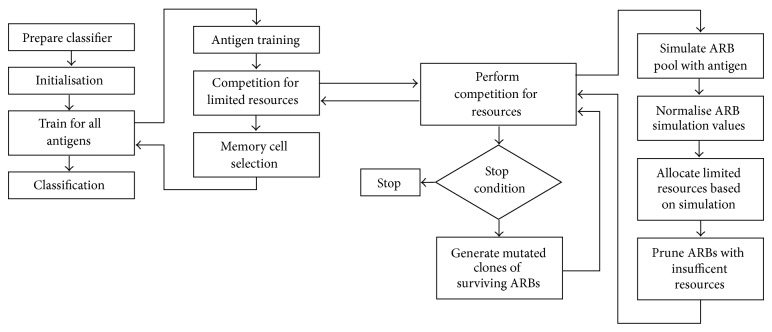
AIRS classification.

The artificial immune-recognition system (AIRS) was implemented by Watkins [12] in his master's thesis. This algorithm is actually a cluster-based implementation of the classifier by using the *k*-nearest neighbor. The AIRS makes generalizations by data reduction; however, the *k*-nearest neighbor uses all of the training data for the classification. Briefly, the AIRS algorithm is about creating a population of artificial recognition balls (ARBs) or memory cells to be representative of the training dataset. The ARB refers to the matching or specific recognition cells. They are populated from mutated clones after an affinity calculation. The ones working best for the purpose of an antigen simulation are selected after competing for limited resources. The AIRS algorithm [13] makes a classification by using the mutated clones of the best match memory for an antigen from the ARBs pool. The algorithm process is given in Figure 4.

This algorithm has three implementations called AIRS1, AIRS2, and Parallel AIRS. There is a persistent resource during the training process in the AIRS1 classifier, whereas AIRS2 uses a temporary resource for each antigen. The AIRS1 also needs a mutation for the class of the generated clones. Another approach on the AIRS2 algorithm is parallelism [14], which is about dividing the dataset into number partitions and processing them individually.

### 3.3. Clonal Selection Algorithms

**Figure 5:**
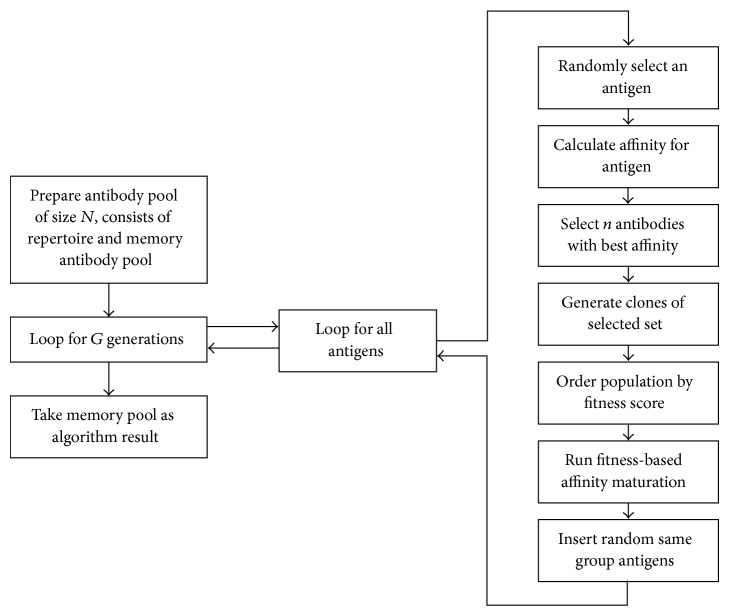
CLONALG algorithm.

Another AIS-based classifier developed by de Castro and Von Zuben [15] is the Clonal Algorithm (CLONALG), which is based on clonal selection theory. Basically, the logic behind this algorithm is that the production of various antibodies, which are generated from B-cells after an antigen simulation, binds to an antigen with a higher value of affinity. These antibodies become trained materials; therefore, they are used to classify new antigens in case of another encounter. The algorithm structure is shown in Figure 5.

**Figure 6:**
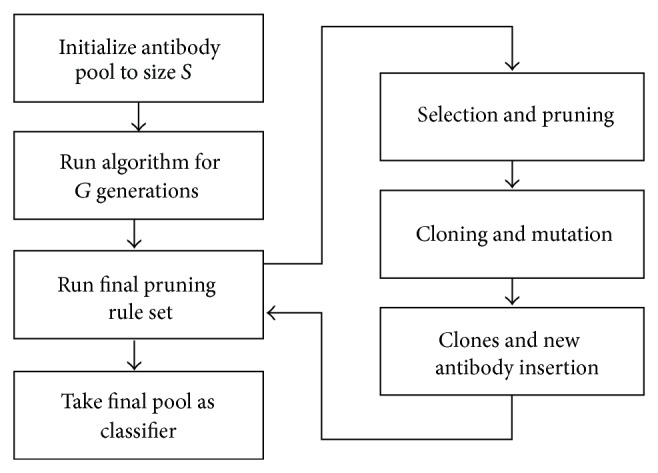
CSCA algorithm.

Another classifier, called the Clonal Selection Classification Algorithm (CSCA) [16], was created by Brownlee. It has an optimization process that maximizes the correctly classified patterns and keeps the incorrect ones minimized. The algorithm process is shown in Figure 6.

## 4. Results

We evaluated 850 cases of headache diagnosis. The dataset included 40 attributes and four classes named “no headache,” “migraine,” “tension type,” and “cluster type” headaches. We used Immunos-1, Immunos-2, Immunos-99, AIRS1, AIRS2, AIRS2-Parallel, CLONALG, and CSCA algorithms. Each type of headache has been analyzed by the algorithms, except some of them, such as 1.5.5 migraine-triggered seizures, childhood periodic syndromes, or other types of primary headaches that are not convenient for survey analysis. Therefore, four patients with hypnic headaches have been eliminated from the analysis. We changed numeric attributes to nominal ones, such as duration, and we collected the duration information at intervals to obtain a better classification. We used a 10-fold cross validation on the dataset to establish reliability through randomization. Each parameter in all of Tables 2, 3, 4, 5, 6, 7, 8, and 9 presented is explained in the appendix.

The Immunos-1 and Immunos-2 algorithms have no user-defined parameters. We used 0.2 for the seed population percentage, which refers to the antigen population percentage in each class for B-cells in order to improve speed and data reduction, 0.5 for the minimum-threshold scalar used for pruning and controlling the population size of the antibodies, and 2 for the total generations, which represents the total number of the refinement iterations for each B-cell population in the Immunos-99 algorithm.

In the AIRS experiment, the stimulation-threshold value, which controls the amount of refinement on ARBs for an antigen, was 0.9 in AIRS1 and 0.5 in both AIRS2 and AIRS2-Parallel. The parameter for the initial memory pool size value was 3 in AIRS1 and 100 in both AIRS2 and AIRS2-Parallel. Other parameters were kept constant for all of the algorithms with the following values: 0.2 for the affinity threshold scalar, which provides a means of affinity between antigens and the training data, 10 for the clonal rate for each of the ARB clones, 2 for the hypermutation rate, which identifies the total mutated clones that are created by a best-matching memory cell, 3 for the number of the nearest neighbors, and “all” for the number of instances to compute the affinity threshold. Additionally, we used four threads for the parallelism in the AIRS2-Parallel algorithm.

We obtained the best results in the CLONALG algorithm with the parameter 30 for the antibody pool size, 0.1 for the clonal factor, 10 for the number of generations, 0.1 for the remaining pool ratio of the total antibodies to allocate to the remaining antibody pool, 1 for the seed, 20 for the selection pool size, which is the total number of antibodies for each antigen exposure in the complete antibody pool, and 2 for the total replacement that is used for the new antibodies stored in the remaining pool. We used the parameter 1 for the number of the nearest neighbors, 0.1 for the clonal scale factor, 50 for the initial population size, 1 for the minimum-fitness threshold, 1 for the number of partitions, 1 for the seed, and 5 for the total generations in the CSCA classifier.

The patients with no headaches could not be diagnosed by the algorithms due to the limited number (only one) of samples having “no headache.” To increase the diagnosis of “no headache” accuracy, more samples are needed.

We reached the best result accuracy of 99.6471% by using the AIRS2-Parallel algorithm. All of the classifiers obtained an accuracy of more than 94% except for the Immunos-2 algorithm which produced the worst result, which means it is not possible to be used for the classification of headaches. The comparison of the algorithms is shown in Table 10.

## 5. Conclusion and Discussion

Computerized diagnosis is one of the important areas in terms of medical applications. These applications [17–19] provide quick and easy information to physicians. This is especially the case with web-based applications [20], which have enabled people to become more connected to each other. Hence, in this study we developed an expert web-based headache-diagnosis system to collect and share patients' data from different cities and used artificial immune system algorithms to classify their headache types. Finally, we examined the diagnostic time and accuracy for the classification of migraine, tension type, and cluster type headaches.

Although the classification performance is based on an algorithm, the density and majority of the data samples are also among the major factors. In the case of our study, the sample size can be mentioned as a limitation, which means that if we had gathered more samples especially for cluster type headaches our algorithm would have run more precisely. This lack of sample variability decreases the likelihood of detecting some headache types, such as migrainous infarction. Also, “probable” headache types are the most complicated types to be diagnosed by doctors because they are related to many different headache types.

Many computer-based headache-diagnosis studies have been conducted with the aim of detecting certain headache types. Researchers have used different methods. For instance, while Pryse-Phillips et al. [4] developed a decision-tree algorithm just for the diagnosis of migraines, we identified all types of primary headaches including migraine. Maizels and Wolfe [21] correctly identified the episodic migraine, tension type, and cluster type headaches. On the other hand, Sarchielli et al. developed an application [22] for primary headaches and also diagnosed chronic headaches [23]. However, Simić et al. [5], who included just 80 participants in their study, made use of rule-based, fuzzy-logic diagnosis software. Although it was successful at diagnosing migraines and tension type headaches similar to our study, it could not diagnose cluster type headaches. In another study, Krawczyk et al. [6] gathered 80% consistency with a study of the various machine learning methods. However, we obtained at least 94% of accuracy, with the best result gathering a 99.65%, excluding the Immunos-2 algorithm.

It can be understood from this study that it is possible to classify headaches by using artificial immune system algorithms. Also, using a web-based diagnosis system is very convenient for patient tracking and information sharing from the physician's point of view.

## Figures and Tables

**Table 1:** Neurologists' headache diagnoses.

Headache types	Number of patients	Percentage %
Migraine	609	71.65%
Tension type	184	21.65%
Cluster type	56	6.59%
No headache	1	0.11%

**Table 2:** Detailed accuracy by class for the Immunos-1 algorithm.

Class	TP rate	FP rate	Precision	Recall	*f*-measure	ROC area	Accuracy
Migraine	0.947	0	1	0.847	0.973	0.974	94.70%
Cluster	0.911	0	1	0.911	0.953	0.955	91.10%
Tension	0.951	0.039	0.871	0.951	0.909	0.956	95.10%
No headache	0	0.025	0	0	0	0.488	0.00%
Weighted avg	0.945	0.008	0.971	0.945	0.957	0.968	94.50%

**Table 3:** Detailed accuracy by class for the Immunos-2 algorithm.

Class	TP rate	FP rate	Precision	Recall	*f*-measure	ROC area	Accuracy
Migraine	1	1	0.716	1	0.835	0.5	100.00%
Cluster	0	0	0	0	0	0.5	0.00%
Tension	0	0	0	0	0	0.5	0.00%
No headache	0	0	0	0	0	0.5	0.00%
Weighted avg	0.716	0.716	0.513	0.716	0.598	0.5	71.60%

**Table 4:** Detailed accuracy by class for Immunos-99 algorithm.

Class	TP rate	FP rate	Precision	Recall	*f*-measure	ROC area	Accuracy
Migraine	0.949	0	1	0.949	0.974	0.975	94.90%
Cluster	0.929	0	1	0.929	0.963	0.964	92.90%
Tension	0.995	0.05	0.847	0.995	0.915	0.973	99.50%
No headache	0	0.005	0	0	0	0.498	0.00%
Weighted avg	0.956	0.011	0.966	0.956	0.959	0.973	95.60%

**Table 5:** Detailed accuracy by class for the AIRS1 algorithm.

Class	TP rate	FP rate	Precision	Recall	*f*-measure	ROC area	Accuracy
Migraine	0.995	0	1	0.995	0.998	0.998	99.50%
Cluster	0.964	0	1	0.964	0.982	0.982	96.40%
Tension	1	0.009	0.968	1	0.984	0.995	100.00%
No headache	0	0	0	0	0	0.5	0.00%
Weighted avg	0.993	0.002	0.992	0.993	0.992	0.995	99.30%

**Table 6:** Detailed accuracy by class for the AIRS2 algorithm.

Class	TP rate	FP rate	Precision	Recall	*f*-measure	ROC area	Accuracy
Migraine	0.995	0.008	0.997	0.995	0.996	0.993	99.50%
Cluster	0.911	0	1	0.911	0.953	0.955	91.10%
Tension	0.995	0.012	0.958	0.995	0.976	0.991	99.50%
No headache	0	0	0	0	0	0.5	0.00%
Weighted avg	0.988	0.009	0.987	0.988	0.988	0.99	98.80%

**Table 7:** Detailed accuracy by class for the AIRS2-Parallel algorithm.

Class	TP rate	FP rate	Precision	Recall	*f*-measure	ROC area	Accuracy
Migraine	0.998	0	1	0.998	0.999	0.999	99.80%
Cluster	0.982	0	1	0.982	0.991	0.991	98.20%
Tension	1	0.005	0.984	1	0.992	0.998	100.00%
No headache	0	0	0	0	0	0.5	0.00%
Weighted avg	0.996	0.001	0.995	0.996	0.996	0.998	99.60%

**Table 8:** Detailed accuracy by class for the CLONALG algorithm.

Class	TP rate	FP rate	Precision	Recall	*f*-measure	ROC area	Accuracy
Migraine	0.998	0.033	0.987	0.998	0.993	0.983	99.80%
Cluster	0.911	0	1	0.911	0.953	0.955	91.10%
Tension	0.978	0.005	0.984	0.978	0.981	0.987	97.80%
No headache	0	0	0	0	0	0.5	0.00%
Weighted avg	0.987	0.025	0.986	0.987	0.986	0.981	98.70%

**Table 9:** Detailed accuracy by class for the CSCA algorithm.

Class	TP rate	FP rate	Precision	Recall	*f*-measure	ROC area	Accuracy
Migraine	0.995	0.008	0.997	0.995	0.996	0.993	99.50%
Cluster	0.982	0	1	0.982	0.991	0.991	98.20%
Tension	0.989	0.008	0.973	0.989	0.981	0.991	98.90%
No headache	0	0	0	0	0	0.5	0.00%
Weighted avg	0.992	0.008	0.991	0.992	0.991	0.992	99.20%

**Table 10:** Overall benchmark results of the algorithms.

Algorithms	Immunos-1	Immunos-2	Immunos-99	AIRS1	AIRS2	AIRS2-Parallel	CLONALG	CSCA
Correctly classified instances	803	609	813	844	840	847	839	843
Accuracy	94.4706%	71.6471%	95.6471%	99.2941%	98.8235%	99.6471%	98.7059%	99.1765%
Incorrectly classified instances	47	241	37	6	10	3	11	7
Inaccuracy	5.5294%	28.3529%	4.3529%	0.7059%	1.1765%	0.3529%	1.2941%	0.8235%
Classification time in seconds	0.05	0.02	0.48	1.12	5.37	12.45	0.81	4.03

## Appendix

- True Positive (TP)
  - Proportion of correctly classified as class *x*/actual total in class *x*
  - Equivalent to Recall
- False Positive (FP)
  Proportion of incorrectly classified as class *x*/actual total of all classes, except *x*
- Precision
  Proportion of the examples which truly have class *x*/total classified as class *x*
- *F*-measure
  - 2 × Precision × Recall/(Precision + Recall),
  - that is, A combined measure for precision and recall
- ROC area (also known as area under curve) is the probability that a randomly chosen positive instance in the test data is ranked above a randomly chosen negative instance.
